# Legal and ethical principles governing the use of artificial intelligence in radiology services in South Africa

**DOI:** 10.1111/dewb.12436

**Published:** 2023-11-27

**Authors:** Irvine Sihlahla, Dusty‐Lee Donnelly, Beverley Townsend, Donrich Thaldar

**Keywords:** artificial intelligence, ethics, law, radiology, software as a medical device

## Abstract

Artificial intelligence (AI) will drastically change the healthcare system. Radiology is one speciality that is most affected as AI algorithms are increasingly used in diagnostic imaging. AI‐enhanced health technologies will, inter alia, increase workflow efficiency, improve diagnostic accuracy, reduce healthcare‐related costs, and help alleviate medical personnel shortages in under‐resourced settings. However, the development of AI‐enhanced technologies in healthcare is fraught with legal, ethical, and human rights concerns. Currently, the use of AI in South African healthcare is not governed by sui generis legislation or ethical guidance focused exclusively and specifically on AI, although various provisions and principles from law and ethics find application. This article outlines these normative principles and explains their relationship with the extant legal obligations and regulatory framework as applied to the use of AI in radiology services in South Africa. The article concludes with three key recommendations for radiology practitioners using AI in South Africa. These are the need for: vigilant monitoring of AI use in practice, reforms to the liability framework, and appropriate guidance from local regulators and the Health Professions Council of South Africa on the ethical use of AI.

## 1 INTRODUCTION

While radiology is rapidly pioneering advances in the use of artificial intelligence (‘AI’) in healthcare, the regulatory policy is struggling to keep pace.^1^ AI systems disrupt the delivery of healthcare services as they operate with varying levels of autonomy and in ways that may equal or surpass human intelligence and ability.^2^ The rapid development and evolution of AI‐enhanced technologies, coupled with a lag in regulatory and ethics initiatives, unmasks crucial legal and ethical gaps.^3^ Moreover, the optimal process for the training of Machine Learning (‘ML’) AI models for radiology is through repeated cycles of learning and implementation, which pose unique challenges to our existing system of regulating medical devices. These are challenges which, if not addressed, are immediate and highly consequential. This article proceeds in three parts. Section 2 defines relevant and important concepts, uses, and risks of AI. Section 3 discusses the legal and ethical principles that underpin the South African clinical healthcare context, and Section 4 concludes with recommendations.

## 2 CONCEPTS, USES, AND RISKS OF AI

### 2.1 Defining artificial intelligence use in healthcare

The Organisation for Economic Co‐operation and Development (OECD) describes AI as:

‘software that aims to emulate human brain intelligence. It is developed with one or more of the techniques and approaches (including but not limited to machine learning, logic, knowledge base, and statistical approaches) and can, for a given set of human‐defined objectives, generate outputs such as content, predictions, recommendations or decisions influencing the environments they interact with’.^4^

The implication is that certain AI software, in particular, that which deploys ML capabilities, can work independently of its developer or end‐user by learning from large amounts of data in a process of continually improving its output. ML enables the system to learn and adapt without following explicit instructions by using models to analyse and draw inferences from patterns in the data itself – thus giving the system the ability to ‘learn’ without explicit programmable instruction.^5^

The United States Food and Drug Administration (FDA) describes two types of algorithms: ‘locked’ and ‘abstract’. This distinction is important as the level of human control, data use and training differs. ‘Locked’ algorithms provide the same output each time the same input is applied and they do not change with use.^6^ They are driven by a predetermined set of instructions – the algorithm – from which the system does not deviate. ‘Abstract’ algorithms have predictive properties, utilise ML functionality, and operate with greater autonomy. These iteratively adaptive AI software systems continually interact with the real‐world environment and may change their output after distribution in the market.^7^ Such software uses ML algorithms, in particular, those that utilise unsupervised, semi‐supervised, and reinforcement learning to operate with varying levels of autonomy.^8^ The algorithmic output from the adaptive AI software using ML may provide different outputs compared to one at the certification stage. Certification of AI software involves the clinical validation of output, concept, effectiveness and safety.^9^

ML systems also present additional safety risks when embedded in certain products used in medical devices, including risks aggravated by faulty design, difficulties stemming from the ML process itself, or flawed, under‐represented or inaccurate data quality and data availability.

Although the legal and ethical risks and uncertainties arising from ‘locked’ and ‘abstract’ algorithms may differ, both require ethical and legal oversight.^10^ In particular, the development of abstract algorithms, through repeated iterations of learning and implementation, challenges extant systems of regulating medical devices.^11^ Accordingly, and in line with such development, medical device regulation and guidance require development to address these risks and to provide institutional arrangements for safe and dependable deployment. This article focuses only on AI systems employing ML – rather than on locked AI software.

A software system should qualify as a medical device before it can be considered Software as a Medical Device (SaMD).^12^ The International Medical Device Regulators Forum (IMDRF) and The Medicines and Related Substances Act 101 of 1965 (‘MRSA’), which principally regulates medical devices in South Africa, similarly stipulate that a ‘medical device’ includes, inter alia, any instrument, apparatus, implement, machine, and software intended by the manufacturer to be used, alone or in combination, *for the diagnosis, prevention, monitoring, treatment or alleviation of disease*.^13^ Thus, a medical device is an instrument that does not achieve its primary purpose through pharmacological, immunological, or metabolic means – but may rely on these mechanisms.^14^ This proviso differentiates a medical device from medicines such as drugs which can be used for similar purposes. As the MRSA and the South African Health Products Regulatory Authority (‘SAHPRA’) guidance document include ‘software’ in the definition of medical devices, a general and purposeful interpretation is that both locked and adaptive AI software are included in the ambit of the provision of medical devices.^15^ Although the MRSA regulates software broadly, and general principles set out in the National Health Act^16^ or other South African health regulations and guidelines will specify standards for consent and professional conduct, no regulations specifically govern the use of AI in the South African healthcare context.

AI software in a medical device includes both software as a medical device (SaMD) and software in a medical device (SiMD).^17^ Whereas SiMD is software for non‐medical purposes, SaMD is AI software embedded in a medical device that serves a medical purpose.^18^ SaMD is a medical device and includes an in‐vitro diagnostic medical device.^19^ It is capable of running on general‐purpose computing platforms and can be used in combination with other medical devices including hardware medical devices.^20^ Furthermore, the software does not meet the definition of SaMD if its purpose is to drive hardware medical devices – that is, it has a non‐medical purpose.^21^ Crucially, the SaMD must have a medical purpose that differentiates it from general‐purpose software that drives hardware medical devices.^22^ Although the local regulation covers software in the definition and risk classification system, it is vague as it does not adequately address differences between the different software types and risk profiles. Furthermore, the regulations classify software in the same classification system as hardware medical devices.^23^ The approach undertaken by the local regulator, the SAHPRA, is different from that of the IMDRF. In contrast, the IMDRF has gone a step further and has provided different risk classification systems for hardware and SaMD,^24^ which the local regulation falls short of. Regulation should strive to eliminate ambiguity,^25^ and hence clear risk classification of AI should be addressed by local policy‐makers and regulatory bodies to provide clarity on AI software as a medical device.

Managing the risk of healthcare user harm requires careful regulatory oversight and consideration of the ethical and legal principles governing the use of AI,^26^ especially for AI software that has novel autonomous capabilities. Naidoo, et al^27^ identify these ethical issues including transparency, privacy, non‐maleficence, accountability, justice and fairness. This article builds on that work and in Section 3 outlines the core ethical principles which are grouped into four themes. It explains their relation to the legal obligations and regulatory framework currently applicable to the use of AI in radiology services in South Africa. This is illustrated by a hypothetical scenario of Transpara^28^ being AI software approved by the FDA for use in digital mammography. This is intended to be used as a concurrent reading aid for physicians interpreting imaging used to identify regions that could be breast cancer.

### 2.2 The use and risks of AI in radiology

AI use in healthcare is becoming ubiquitous – affecting all aspects.^29^ In 2018, the FDA approved the first AI software for medical use^30^ and since then has approved more than 350 SaMD with more than half of the AI software being intended for use in radiology.^31^ Likewise, isolated cases of AI use in South African radiology services and healthcare start‐ups have been reported.^32^ The vast amount of health data generated from the use of AI in radiology has the potential for future health research and clinical application.^33^ The benefits of the clinical application of AI in radiology are numerous as it can simplify ordering, scheduling, protocoling, image acquisition, image interpretation, reporting, communication, and billing.^34^ AI is incorporated into a clinical radiology workflow ranging from administrative purposes, imaging acquisitions, image pre‐processing, segmentation, reporting, and helping with diagnosis.^35^ Image‐based diagnostic use of AI in a radiology practice involves detection, characterisation and quantification analysis of pathology.^36^ For example, AI can be used in the staging of cancer and monitoring treatment outcomes.^37^ Therefore, AI use can result in increased workflow efficiency, reduced diagnostic errors, and improved accuracy.^38^ In each of these contexts, AI holds great promise for advances in the efficiency and quality of healthcare. However, despite the manifest potential benefits, healthcare remains a high‐risk application of AI, especially for those cases where the algorithmic output from autonomous AI has a significant impact on healthcare user management, treatment, diagnosis, and prognosis – which can lead to irreparable harm or even death.

The risk of harm from the use of AI may include physical harm to the healthcare user and non‐physical or rights‐based harm. For example, unauthorised access to a healthcare user's health data will result in non‐physical harm. The malevolent actor who obtained the confidential health records may publish embarrassing or defamatory information about the healthcare user. As a result, the healthcare user's right to confidentiality provided in section 14 of the National Health Act^39^ and common law rights of *dignitas* (dignity) and *fama* (good name and reputation) are harmed – even if the healthcare user does not suffer any physical bodily harm. On the other hand, a malevolent actor may hypothetically insert malicious code into the software that is intended to disrupt the operation of a medical device and cause direct physical harm.^40^ Physical harm from the use of AI can also arise when the software drifts away from its intended use or provides inaccurate algorithmic output.^41^ In addition, harm to the healthcare user can arise from many other causes, including issues of data quality arising from flawed, biased, or under‐representative data used to train the algorithm or faulty design.^42^ Notwithstanding the vast potential uses of AI in healthcare, significant ethical and legal harm can, however, arise from its use.

## 3 LEGAL AND ETHICAL PRINCIPLES

### 3.1 Ethical and legal issues

The autonomous capabilities of some AI software imply that nobody is in full control of AI's algorithmic output at all times. Hence, as algorithms become more technically advanced and attain higher levels of autonomy, it becomes more difficult to adequately demonstrate how AI reaches its decisions, both ex‐ante in a regulatory approval application and ex post facto in litigation or disciplinary inquiries following harm to a healthcare user. Therefore, AI software with autonomous capabilities presents unique challenges to the process of ascribing legal blame^43^ and, as a result, exposes legal gaps when autonomous AI is used.^44^ Human oversight through governance mechanisms that include humans in the decision‐making loop is one of the ways that help ensure that fundamental human rights are not undermined during autonomous AI use.^45^ The four ethical–legal challenges are now discussed.

### 3.2 Transparency and the requirement of informed consent

Transparency is described in AI ethics literature as a principle where an AI decision should always be discoverable and addresses explainability, traceability, and interpretability.^46^ The concept of transparency incorporates the requirement of explainability, which means that there should be a disclosure of how the AI system functions.^47^ However, implementation of the obligation of transparency could be challenging considering the black box effect of AI.^48^ The black box effect arises from the fact that the functionality of AI is too complex for a human to comprehend.^49^ Additionally, it occurs as a mechanism for protecting proprietary rights put in place by the AI developer.^50^ Consequently, providing full disclosure to the healthcare user about how the system operates may be problematic in the current state of AI development. Alternatively and practically, it may be feasible to provide sufficient information to the healthcare user. To circumvent the opacity and complex nature associated with AI, propositions have been made to introduce interpretable models to provide explanations of algorithmic output and ensure transparency.^51^ The models aim to provide accurate and trustworthy explanations in non‐technical terms to health personnel and healthcare users.^52^ This will reinforce the statutory obligation to provide transparency and accountability since complete explanations are not feasible. The statutory obligation emanates from the right of access to information in section 32 of the Constitution,^53^ the Promotion of Access to Information Act,^54^ section 24 of the Consumer Protection Act,^55^ and section 71 of the Protection of Personal Information Act (‘POPIA’).^56^ Interpretable models can help provide sufficient information about the AI software to the end users, which can then be used to obtain informed consent which is pivoted on the provision of sufficient information to the healthcare user.

The healthcare user–radiologist relationship is rooted in informed consent and therefore the radiologist has an ethical and legal duty to inform and disclose to healthcare users how the AI software operates and reaches its decisions. Informed consent is therefore linked to the obligation of transparency – as both involve the disclosure of information to healthcare users. The HPCSA guidelines on informed consent stipulate that the clinician should provide sufficient information to healthcare users in a manner that they understand so that they can exercise their right to make informed decisions about their care.^57^ Moreover, the disclosure should use plain and simple language to enable those adversely affected by the AI software to challenge its output and the logic which underlies the algorithmic decision.^58^ Sufficient information encompasses the range of diagnostic procedures and treatments available to the healthcare user and the benefits, risks and consequences related to each of the available options.^59^ The courts have also held that sufficient information should involve the whole transaction, the material risk, and foreseeable consequences.^60^ AI use in radiology will fall under the range of diagnostic and treatment procedures available to the healthcare user, and hence the clinician is obliged to provide sufficient information to the healthcare user.

Section 6 and 7 of the National Health Act^61^ and the HPCSA ethical guidelines^62^ stipulate that the healthcare practitioner should obtain informed consent from the healthcare user. Sections 11 and 18 of POPIA further reinforce the requirement of consent as it provides that personal information that includes health data be collected after voluntary, specific and informed consent has been obtained and the healthcare user should be notified about the collection of personal information.^63^ In obtaining informed consent, the radiologist will be required to explain to healthcare users, using non‐technical terms, the function or purpose of the AI software – rather than the intrinsic complex processes of the AI software. In *Christian Lawyers Association v Minister of Health,* 64 the court held that informed consent is based on the three elements of knowledge, appreciation, and consent. Thus, the fiduciary duty of the healthcare practitioner to obtain informed consent by providing sufficient information to the healthcare user needs to be fulfilled in a manner consistent with statutory and common law requirements. This information can be made available by the AI software manufacturer to the clinician so that they can provide informed consent appropriately. For example, Transpara describes the indications for use, the target population, how the AI software operates, and the required level of expertise needed to understand the AI software output in non‐technical language that healthcare users can understand.^65^

### 3.3 Data protection

The privacy of healthcare users’ health data should be protected during the use of AI software in healthcare.^66^ Personal health data use by AI involves the collection, sharing, archiving, and disposal of such data. There is an important need for vigilant data management and security systems to be in place to ensure that unauthorised data access is not permitted. Sections 19, 20, 21 and 22 of POPIA provide that security measures should be in place to protect the integrity and confidentiality of personal information.^67^ The responsible party, inter alia, the hospital, private radiology practice or group hospital, should take appropriate and reasonable technical measures to guard against loss, damage, or unlawful access to personal information. The health establishment should be proactive in identifying internal and external risks that may compromise the personal information of healthcare users in their care and should have appropriate safeguards in place which are updated continually. Section 22 of POPIA further requires that personal information should be kept confidential, must not be disclosed, and the data subject and information regulator should be notified in the case of a data breach.^68^ Furthermore, in the unfortunate event of a data breach, the health establishment should provide the healthcare user with sufficient information that will allow the data subject to take preventive measures.^69^ Therefore, the radiology practice is obliged to take vigilant steps to protect healthcare users’ sensitive health data in order to uphold healthcare users' privacy and dignity.

In addition to the statutory requirement to protect the privacy of healthcare users, AI software use also requires adherence to the notion of ‘privacy by design’ as mandated by privacy legislation – to ensure that privacy protection is ‘built‐in’ to the design of AI software to minimise the collection of personal data and the risks of unintended privacy harm.^70^ Failure to observe the aforementioned statutory tenets in POPIA can result in health data being accessed without the healthcare user's consent, which can infringe on their informational privacy as previously observed in the NHS–Deepmind partnership.^71^ Failure to protect healthcare user privacy and to fully comply with data protection laws is unlawful and also erodes public trust in AI. This may result in hesitancy and reduced uptake of AI in the health sector. In addition, it could embroil health practitioners in litigation. Hence, it is vital to uphold healthcare user privacy with all use of AI software in radiology. The World Health Organisation (WHO) recommends the use of a data protection impact assessment before selecting or using AI software in healthcare to minimise harm from data privacy breaches and to ensure that healthcare user data use adheres to international standards.^72^

### 3.4 Non‐maleficence: Liability – what happens when things go wrong?

A core principle in AI ethics is *non‐maleficence*, which according to Beauchamp and Childress means ‘one ought not to inflict harm or evil’.^73^ Thus, it is defined as a moral duty to avoid the harm that can arise from any action.^74^ This has added significance in the health sector, where practitioners risk liability if they cannot comply with the obligation to ‘do no harm’ to healthcare users, which is grounded in the Hippocratic oath^75^ and the ethical–legal duties imposed by law.^76^ Therefore, procedures for risk assessment and measures to prevent harm should be adopted.^77^ AI software must be robust, secure and safe to avoid an unreasonable risk of harm.^78^ The safety of AI software must be guaranteed throughout its lifecycle. We propose that in the context of radiology services, these safety outcomes can be achieved by adopting similar principles to the ALARA principle which is widely used in radiology. ALARA is an acronym for ‘As Low As Reasonably Achievable’ and requires that harm accruing from radiation during imaging should be kept as low as possible at all times.^79^ In essence, it is an operationalisation of the principle of non‐maleficence and, by analogy, a similar principle should be applied to keep the AI software safe within legally acceptable risks of healthcare user harm, while benefitting from its use in healthcare.

The risk of healthcare user harm and health practitioner liability can be illustrated by considering a hypothetical scenario that could arise from the use of Transpara installed in a radiology practice. Transpara is intended to be used as a concurrent reading aid for physicians interpreting imaging used to diagnose breast cancer. It identifies regions that could be breast cancer based on the images, assesses their likelihood of being cancer, and gives a recommendation to the radiologist. In our hypothetical scenario, a healthcare user's imaging is interpreted by the software and its output is that a certain mass is highly unlikely to be cancer. Acting on the recommendation, the radiologist does not take any further action. Suppose, however, that three years later the healthcare user presents again to the healthcare facility and the same mass is subsequently confirmed as advanced breast cancer.

The important question that will arise from the above hypothetical scenario is who or what is at fault, and who – if anyone at all – can be held legally liable in delict by the healthcare user? Is it the AI developer, the hospital that purchased the AI software, the autonomous AI software, the radiologist, or all of these actors, or is nobody liable?

Not all harm that arises from healthcare use results in liability. Liability is established only if certain requirements – over and above the harm itself – are met. What these requirements are would depend on how the harmed healthcare user frames his/her potential lawsuit. The healthcare user in our hypothetical scenario can potentially base her action on common law delictual liability, on the statutory provisions regarding product liability in the Consumer Protection Act, or on both. In the case of delictual liability, she would need to prove that the healthcare practitioner acted negligently, and in the case of the Consumer Protection Act (CPA), she would need to prove that there was a defect in the AI software.^80^

We now explore these two avenues of establishing liability in more detail.

The common law applies the principle of fault‐based liability to medical professionals. This requires one to determine whether (at the time of the incident) the radiologist acted as a reasonable member of his or her profession. It is reasonable to expect the radiologist to independently apply his or her mind to the assessment of the images. Furthermore, in each case, it is a question of fact whether the skilled eye of a radiologist would reasonably have identified suspicious pathology in the image and taken further steps to investigate the mass. If these steps would have been taken by a reasonable radiologist and were not taken by the treating radiologist, then fault (in the form of negligence) is established. However, what would a reasonable radiologist do if an AI system that is trained on thousands of images indicates that a certain mass that may seem to be suspicious pathology is in fact highly unlikely to be cancer – as in our hypothetical scenario? Would a reasonable radiologist ignore the AI system's advice and investigate the suspicious mass? Or would a reasonable radiologist accept the AI system's advice based on the fact that the AI system is trained on far more data than what any human could ever comprehend? There is of course no case law to answer this question – as yet. However, as the use of AI systems becomes increasingly common and accepted in healthcare practice, we suggest that the courts are likely to gradually lean towards accepting that a reasonable healthcare practitioner can accept an AI system's advice – perhaps with an exception in cases of obvious mistakes. After all, what is the use of an AI system in healthcare practice if healthcare practitioners cannot rely on it to be more accurate than humans? However, at present there is no guidance in case law on how to apply principles of fault‐based liability in a scenario where the outcome is primarily attributable to an error by an AI system that could not reasonably have been identified by the healthcare practitioner operating the system, nor anticipated by the developer. Accordingly, determining negligence when AI is used in healthcare will be a complex process.^81^

In terms of section 61 of the CPA, the practitioner or establishment offering radiology services using AI software would be regarded as the supplier of the AI product in relation to the healthcare user.^82^ The CPA in section 61(1) establishes a form of strict liability for harm caused by the supply of *unsafe* goods.^83^ According to section 53, the definition of *unsafe* requires that ‘due to a characteristic, failure, defect or hazard, particular goods present an extreme risk of personal injury or property damage to the consumer or other persons’. Given the requirement of *extreme* risk, it is doubtful whether a well‐trained AI system would qualify as *unsafe* goods. Also, the health user has to demonstrate that the harm arose because of the defect in the AI system. The claimant must prove harm which is causally related to a wrongdoing/defect of AI as part of the delictual requirements. However, demonstrating the defect with AI will be difficult, in particular, because of the inherent opacity and the complex nature of how the AI reaches its decision. This is vital as the healthcare user is required by the CPA to prove that the harm was wholly or partially a result of a product defect. In obiter, the court in *Motus Corporation and another vs Wentel* said that ‘Not every small fault is a defect in terms of CPA’.^84^ Thus the healthcare user has to prove the defect to be material. Satisfying this burden of proof can be challenging.^85^ Consequently, the healthcare user may be left with no legal recourse from the CPA.

Moreover, a healthcare practitioner or hospital can rely on section 61(4)(c) of the CPA to escape strict liability if the healthcare practitioner or hospital could not reasonably have been expected to discover the defect.^86^ In addition, the radiologist can rely on section 61(4)(a) of CPA to escape liability if the AI system complied with any public regulation which prescribes the standards, certification and registration of the AI system in South Africa.

Some AI developers and healthcare facilities may opt to use contractual clauses to minimise liability arising from the use of AI software. Contractual exemption clauses excluding liability for harm, unless it is caused intentionally or through gross negligence, remain valid but highly controversial.^87^ Regrettably, allowing contractual clauses to limit liability would offer less protection to healthcare users, be detrimental to the interest of all parties, and erode public trust.^88^ The clauses limiting all liability for harm would be contrary to the codified provisions of sections 48(1)(c) and 51(1)(c)(i) of the CPA as they would be unfair, unreasonable or unjust to the healthcare user.^89^ It would be grossly unfair, unjust and contrary to the norm of good faith to provide health services that lead to harm but do not offer redress to the healthcare user. Moreover, it would undermine the constitutional right of access to healthcare services in section 27 of the Constitution.^90^

It will be difficult and uncertain for a healthcare user who was harmed in our hypothetical scenario to establish liability and be able to successfully claim from anyone involved. Such ambiguities, therefore, strengthen the urgent need for *sui generis* AI legislation to address liability for harm and to provide paramount legal certainty in a manner that strikes the necessary balance between public benefit from technological innovation, healthcare user safety, and privacy concerns.^91^

The hypothetical case and the dilemma within the existing liability regiments are strong cases for advocating strict liability for AI in *sui generis* AI legislation. The *sui generis* legislation will need to clearly outline the standards that AI should adhere to and the evidentiary elements required to prove liability arising from AI use. Ethical principles must be transformed into statutory provisions to provide legal enforcement.^92^ Therefore, the *sui generis* AI legislation will enhance the protection of healthcare users’ rights, improve beneficence, minimise maleficence and provide for accountability which will enhance public trust in AI. In the context of the CPA, a defect must be proven, and in the AI context, non‐compliance with a fundamental ethics principle would be required to trigger strict liability. Such a strict liability framework can be paired with a mandatory public insurance scheme to provide for compensation, rehabilitation and restitution.^93^ The redress afforded through the insurance scheme will be on a no‐fault basis connected to a reconciliatory‐based mechanism grounded in Ubuntu, which promotes accountability.^94^ Consequently, the healthcare user will be afforded a redress mechanism through the no‐fault basis while the reconciliatory process gives the healthcare system a platform to reflect and learn from the case – in turn harnessing valuable recommendations to prevent re‐occurrence in the future.

Different propositions for liability in AI have been suggested.^95^ Soyer and Tettenborn broadly propose two options.^96^ One is to allow the law to gradually deal with AI technology,^97^ which can include a common law case‐by‐case basis. Alternatively, they propose creating a different novel liability framework. The framework can be a combination of fault‐based liability, strict liability, or a hybrid of both. The hybrid liability will depend on whether the application is a high risk, medium risk or low risk, and also on the particular risk attached to the use of AI.^98^ This categorisation of AI software into different risk groups is consistent with the approach proposed by the IMDRF and the European Union and will be useful in determining liability.^99^

The envisioned accountability framework will improve public trust in AI systems while stimulating further innovation and development of AI. Furthermore, public policy considerations may outweigh individual healthcare users’ rights to claim legal recourse against AI developers. The government can develop a public policy instrument to deal with legal claims related to AI, akin to the counter‐measures insurance programme undertaken by the US government during the COVID pandemic to minimise legal claims against vaccine producers.^100^ Lastly, punitive sanctions by the local regulator may in exceptional cases be used against AI developers who produce software that results in serious harm and infringes the safety of the healthcare user.^101^

### 3.5 Accountability, justice and fairness

In the event of harm arising from AI use, mechanisms should be in place to ensure that proper accountability can be achieved. Regulators, including the SANAS and SAHPRA, will play a crucial role in ensuring that AI products are deployed only when they conform to standards that provide for safe and efficacious use in the healthcare sector and that their performance in clinical settings is monitored by an effective reporting system and clinical audits.^102^ This will also help with post‐deployment regulatory monitoring as mandated by the local health product regulator. Although it is difficult to achieve, the process should provide mechanisms to trace the AI algorithmic output, as it is vital to precisely pinpoint the area where the problem might have arisen in the event of harm arising from AI use and to help with assigning legal liability. This will be valuable in helping practitioners to explain to healthcare users the possible risks and mitigating factors in place during AI software use – thus strengthening transparency and fortifying trust in AI software in healthcare.

Algorithmic bias and unfair discrimination are additional challenges posed by AI and can be detrimental. This will be contrary to Section 9 of the Constitution which prohibits unfair discrimination.^103^ Bias is a systematic difference in treatment, observation, perception, representation, prediction or decision of certain objects, people or groups of people in comparison to others.^104^ Discrimination is ‘any act or omission which imposes any burden, obligation or disadvantage either directly or indirectly on any person’.^105^

Algorithmic injustices in AI through bias and discrimination occur as most of the available training dataset is from a circumscribed population which excludes minority populations.^106^ With informational asymmetry because of selection bias, AI software used in healthcare can potentiate and deepen social disparities which already exist in society.^107^ Obermeyer et al demonstrated that an AI algorithm used to manage and refer healthcare users with complex diseases for further management exhibited racial bias.^108^ Similarly, it has been shown that AI can determine the race of an individual from radiology images through a process that does not depend on common anatomical or observable character traits such as height, skin colour, or hair colour,^109^ and it will be difficult to detect or remove the bias from the AI system.^110^ Therefore, to minimise bias and allow generalisability, the training and testing datasets should include participants from an inclusive diverse group which encompasses the population on which the AI software will be used in clinical practice.

The goal of AI in radiology should be to extract value from its use through just and beneficial outcomes rather than deepening ethical and societal imbalances.^111^ The WHO ethical guidelines have been proposed to guard against healthcare user harm and have made numerous key recommendations which include inclusive participation by all stakeholders during the design phase, the stakeholder having sufficient understanding of the task the AI software is supposed to perform, and the conditions necessary to ensure it performs the task accurately.^112^ In addition, AI developers should ensure that AI systems perform well‐defined tasks with accuracy and reliability.^113^ The authors identify four ethical–legal red flags for radiologists: liability, data security, privacy, and bias and discrimination of AI software.

Low to Middle Income Countries (LMICs) including South Africa are ladened with another layer of constraints above and beyond the legal and ethical setbacks outlined above. These hindrances encompass a lack of hardware and computing resources, a lack of specialised personnel and undeveloped digital health systems.^114^ LMICs often lack structural facilities to develop and maintain digital health systems.^115^ Digital health is the cornerstone upon which electronic data is created, stored, annotated and curated for use in AI development and training. Robust digital health systems require hardware and computing powers to be fully functional and provide high‐quality digital health datasets. Electronic health records which are components of the broader digital health landscape are sporadically put into practice within South Africa's healthcare system. This limited adoption is primarily due to a shortage of financial resources, a lack of skilled personnel, and inadequate infrastructure.^116^ Consequently, the locally available health data is typically not in a format that is readily useable in AI development.

High‐quality digital health datasets are vital for the development of AI in healthcare.^117^ In general, the accuracy of the algorithmic output is intricately related to the quality of the data that is used to train the algorithm. One of the critical factors that impede the development of AI in African healthcare systems is the lack of large clinical quality datasets for the training, development and validation of AI.^118^ Furthermore, the health datasets used to train and validate AI algorithms require to be annotated and labelled by specialised experts and this is curtailed by a lack of skilled personnel. As a result of these compounding challenges, AI algorithm development and implementation in LMICs are likely not to reach their full potential compared to high‐resourced settings.^119^ Thus AI development in LMICs healthcare systems is still in its embryonic phases with development gaps in LMICs in comparison with high‐income countries (HICs).

The development gaps in AI that exist within LMICs affect the deployment of AI algorithms. In HICs, AI systems are set to provide support to highly skilled healthcare practitioners whereas in LMICs, due to a lack of medical specialists, AI will be used to provide this highly specialised knowledge to the populace. Therefore using an AI developed in HICs countries may be challenging and must be subject to a robust evaluation of safety and efficacy in LMICs considering the different health needs of the local populace and the available healthcare infrastructure and resources.^120^ Additionally, AI developed in HICs might contain bias and prejudice inherent in the testing data population. Deploying such AI software without a comprehensive safety and efficacy assurance case in the local context can perpetuate algorithmic bias. Furthermore, AI algorithms developed in HICs can be affected by differences in disease prevalence when deployed in LMICs with a decrease in accuracy and sensitivity.^121^ The change in accuracy will result in algorithmic output which may not be correct. This can potentially result in misdiagnosis and harm to healthcare users thus infringing upon their health and safety. The shortcomings of the SAHPRA, the local regulator discussed above illustrate that even where a mature regulator exists, it may still have inadequate frameworks in place to effectively deal with AI medical devices.

## 4 RECOMMENDATIONS FOR RADIOLOGY PRACTICE

The use of AI in low‐resourced health systems, including South Africa, holds great promise for improving health outcomes. The huge potential of AI is, however, associated with intricate ethical–legal issues. First, we recommend that local radiology practitioners together with SAHPRA should implement vigilant post‐deployment monitoring, clinical audits, and silent clinical trials^122^ to check for accuracy, safety and reliability. In addition, SAHPRA can also allow the use of AI software in regulatory sandboxes.^123^ Regulatory sandboxes allow the use of innovative AI software in specialised healthcare centres while under the supervision of the regulator, and presents an opportunity to undertake both ethical and human rights impact assessments before widespread deployment in the local healthcare systems.^124^ Secondly, adequate liability cover should be in place to address potential liability arising from AI use. Thirdly, professional bodies, including the HPCSA and the RSSA, should provide updated ethical guidance for radiology practices on standards to be adhered to during AI use, which are similar to initiatives embarked upon by other leading societies.^125^ Additionally, there is a need for capacity building,^126^ global collaboration, personnel training and phased introduction of AI in LMICs with adherence to local data rights and socio‐cultural factors.^127^

## 5 CONCLUSION

There is a strong and complementary relationship between ethical principles and legislative provisions which provide foundational guidance to medical treatment. AI is challenging these pre‐existing norms. However, it remains to be seen if these pre‐existing norms are adequate to fully cover the operational use of AI in South African radiology services. This is critical considering the disruptive nature of AI and the unique ethical and legal challenges it poses. This heightens the call for the promulgation of a *sui generis* AI legislation to cover the development, certification and deployment of responsible and ethical AI in the South African healthcare system. Furthermore, there is a need for capacity building, the addition of local socio‐cultural strategies and global collaboration with other regulators and regulatory bodies to improve the acceptance and assimilation of AI medical devices.

## AUTHORSHIP

Irvine Sihlahla worked on the project conceptualisation, literature search, writing of the original draft, and revision. Dusty‐Lee Donnelly, Beverley Townsend, and Donrich Thaldar all worked on the conceptualisation, revision, and supervision. All authors were responsible for the manuscript editing and approval of the final version.

## CONFLICT OF INTEREST STATEMENT

### 1 Ethics approval and consent to participate

Not applicable.

### 2 Consent for publication

Not applicable.

### 3 Availability of data and materials

Not applicable.

## COMPETING INTERESTS

All authors have no competing financial and non‐financial interests related to the present paper to disclose.

## Footnotes

1 Recht, M.P., Dewey, M., Dreyer, K., Langlotz, C., 4, Wiro Niessen, W., Prainsack, B., & Smith, J.J. (2020). Integrating artificial intelligence into the clinical practice of radiology: Challenges and recommendations. European Radiology. 30(6):3576‐3584; Pesapane, F.,Volonté, C.,Codari, M., & Sardanelli, F. (2018). Artificial intelligence as a medical device in radiology: Ethical and regulatory issues in Europe and the United States. Insights into Imaging. 9(5):745‐53.

2 Organisation for Economic Co‐operation and Development (OECD). (2019). Recommendation of the Council on Artificial Intelligence. Retrieved October 28, 2022, from https://legalinstruments.oecd.org/en/instruments/OECD-LEGAL-0449.

3 Pesapane, et al., op. cit. note 1(ii), p.747.

4 OECD, op. cit. note 2, p.7.

5 International Medical Device Regulatory Forum (IMDRF). (2022). Machine Learning‐enabled Medical Devices: Key Terms and Definitions. Retrieved November 29, 2022, from https://www.imdrf.org/documents/machine-learning-enabled-medical-devices-key-terms-and-definitions

6 US Food and Drug Administration (USFDA). (2019). Proposed Regulatory Framework for Modifications to Artificial Intelligence/Machine Learning (AI/ML) Based Software as a Medical Device (SaMD). Retrieved September 18, 2022, from https://www.google.com/url?sa=t&rct=j&q=&esrc=s&source=web&cd=&cad=rja&uact=8&ved=2ahUKEwjT3_GR1Pf3AhXBolwKHdm6CRYQFnoECBAQAQ&url=https%3A%2F%2FError! Hyperlink reference not valid.

7 Ibid: 3.

8 Mahesh, B. (2020). Machine learning algorithms – a review. International Journal of Science and Research. 9:381‐386.

9 International Medical Device Regulatory Forum. (2017). Software as a Medical Device (SaMD): Clinical Evaluation.Retrieved August 18, 2022, from https://www.imdrf.org/documents/software-medical-device-samd-clinical-evaluation.

10 Minssen, T., Gerke, S., Aboy, M., Price II, W.N., & Cohen, I.G. (2020). Regulatory responses to medical machine learning. Journal of Law and the Biosciences. 7(1):1‐18; Vayena, E., Blasimme, A., & Cohen, I.G. (2018). Machine learning in medicine: Addressing ethical challenges. PLOS Medicine. 15(11):e1002689.

11 USFDA, op. cit. note 6, p.3; Gilbert, S., Fenech, M., Hirsch, M., Upadhyay, S., Biasiucci, A., & Starlinger, J. (2021). Algorithm change protocols in the regulation of adaptive machine learning‐based medical devices. Journal of Medical Internet Research. 23(10):e30545.

12 IMDRF, op. cit. note 5, p.5; International Medical Device Regulatory Forum. (2013). Software as a Medical Device (SaMD): Key definitions. Retrieved May 10, 2022, from http://www.imdrf.org/docs/imdrf/final/technical/imdrf-tech-131209-samd-key-definitions-140901.pdf.

13 Ibid: 6; Republic of South Africa. (1965). Medicines and Related Substances Act No. 101 of 1965. Retrieved November 18, 2022, from https://www.gov.za/documents/drugs-control-act-7-jul-1965-0000.

14 Townsend, B.A. (2020). Software as a medical device: Critical rights issues regarding artificial intelligence software‐based health technologies in South Africa. Journal of South African Law/Tydskrif vir die Suid‐Afrikaanse Reg. (4):747‐762.

15 Ibid: 749; South African Health Product Regulatory Authority. (2023). Classification of medical devices and IVDs. Retrieved July 07, 2023, from https://www.sahpra.org.za/document/guideline-for-classification-of-medical-devices-and-ivds/.

16 Republic of South Africa. (2003) National Health Act 61 of 2003. Retrieved May 16, 2023, from https://www.gov.za/documents/national-health-act .

17 International Telecommunication Union. (2022). Good practices for health applications of machine learning: Considerations for manufacturers and regulators. Retrieved March 3, 2023, from https://www.itu.int/en/ITU-T/focusgroups/ai4h/Documents/del/DEL2_2-A20220922-%28Prepub%29.pdf.

18 Ibid: 4.

19 IMDRF, op. cit. note 5, p.6.

20 Ibid: 6.

21 Ibid: 6.

22 Townsend, op. cit. note 14, p.759.

23 Republic of South Africa. (2016). Medicines and Related Substances Act No. 101 of 1965. Regulations Relating to Medical Devices and In‐vitro Diagnostic Medical Devices. Government Gazette No. 1515:40480. Retrieved February 21, 2023, from https://www.gov.za/documents/medicines-and-related-substances-act-regulations-medical-devices-and-vitro-diagnostic-2.

24 IMDRF, op. cit. note 5, p. 4; International Medical Device Regulatory Forum. (2014). Software as a medical device: Possible framework for risk categorization and corresponding considerations. Retrieved August 18, 2022, from https://www.imdrf.org/documents/software-medical-device-possible-framework-risk-categorization-and-corresponding-considerations.

25 Pesapane, et al., op. cit. note1, p.747.

26 Morley, J., Machado Caio, C.V., Burr, C., Cowls, J., Joshi, I., Taddeo, M., & Floridi, L. (2020). The ethics of AI in health care: A mapping review. Social Science & Medicine. 260:113172:1‐14.

27 Naidoo, S., Bottomley, D., Naidoo, M., Donnelly, D., & Thaldar, D. (2022). Artificial intelligence in healthcare: Proposals for policy development in South Africa. South African Journal of Bioethics and Law. 15(1):11.

28 American College of Radiology Data Science Institute. (2022). AI Central. Retrieved November 20, 2023, from https://aicentral.acrdsi.org/.

29 Matheny, M.E., Whicher, D., & Israni, S.T. (2020). Artificial intelligence in health care: a report from the National Academy of Medicine. JAMA. 323(6):509‐510; Benjamens, S., Dhunnoo, P., & Meskó, B. (2020). The state of artificial intelligence‐based FDA‐approved medical devices and algorithms: An online database. NPJ Digital Medicine. 3(1).

30 Arndt, R. (2018 April 12). FDA grants first approval to autonomous AI‐powered medical device. Modern Healthcare. Retrieved June 26, 2022, from https://www.modernhealthcare.com/article/20180412/NEWS/180419969/fda-grants-first-approval-to-autonomous-ai-powered-medical-device.

31 Benjamens, et al., op. cit. note 29, p.2; US Food and Drug Administration. (2022). Artificial intelligence and machine learning (AI/ML)‐enabled medical devices. Retrieved November 5, 2022, from https://www.fda.gov/medical-devices/software-medical-device-samd/artificial-intelligence-and-machine-learning-aiml-enabled-medical-devices?utm_source=FDALinkedin#resources.

32 Philips Foundation. (2021). Philips Foundation deploys AI software in South Africa to detect and monitor COVID‐19 using chest X‐rays. Retrieved September 11, 2022, from https://www.philips-foundation.com/a-w/articles/CAD4COVID.html; Tracxn. (2022). AI in healthcare startups in South Africa. Retrieved November 24, 2022, from https://tracxn.com/explore/AI-in-Healthcare-Startups-in-South-Africa.

33 Pesapane, et al., op. cit. note1, p.747.

34 Noguerol, T.M., Paulano‐Godino, F., Martín‐Valdivia, M.T., Menias, C.O., & Luna, A. (2019). Strengths, weaknesses, opportunities, and threats analysis of artificial intelligence and machine learning applications in radiology. Journal of the American College of Radiology. 16(9 Pt B):1239‐1247; Rezazade Mehrizi, M.H., van Ooijen, P. & Homan, M. (2021). Applications of artificial intelligence (AI) in diagnostic radiology: A technography study. European Radiology. 31(4):1805‐1811.

35 Hosny, A., Parmar, C., Quackenbush, J., Schwartz, L.H., & Aerts, H.J.W.L. (2018). Artificial intelligence in radiology. Nature Reviews. Cancer. 18(8):500‐510.

36 Bi, W.L, Hosny, A., Schabath, M.B., Giger, M.L., Birkbak, N.J., Mehrtash, A., et al. (2019). Artificial intelligence in cancer imaging: Clinical challenges and applications. CA: A Cancer Journal for Clinicians. 69(2):127‐157; Fazal, M.I., Patel, M.E., Tye, J., & Gupta, Y. (2018). The past, present and future role of artificial intelligence in imaging. European Journal of Radiology. 105:246‐250; Bohr, A., & Memarzadeh, K. (2020). The rise of artificial intelligence in healthcare applications. Artificial Intelligence in Healthcare. 25‐60.

37 Bi, et al., op. cit. note 36, p.129‐136.

38 Topol, E.J. (2019). High‐performance medicine: The convergence of human and artificial intelligence. Nature Medicine. 25(1):44‐56; Botwe, B., Akudjedu, T., Antwi, W., Rockson, P., Mkoloma, S.S., Balogun, E.O., et al. (2021). The integration of artificial intelligence in medical imaging practice: Perspectives of African radiographers. Radiography. 27(3):861‐866.

39 National Health Act 61 of 2003, op. cit. note 16.

40 Finlayson, S.G., Chung, H.W., Kohane, I.S., & Beam, A.L. (2018). Adversarial attacks against medical deep learning systems. arXiv. arXiv:180405296.

41 Iwashita, A.S., & Papa, J.P. (2018). An overview on concept drift learning. IEEE Access. 7:1532‐1538; Sahiner, B., Chen, W., Samala, R.K., & Petrick, N. (2023). Data drift in medical machine learning: Implications and potential remedies. British Journal of Radiology. 96(1150):20220878.

42 Townsend, op. cit. note 14, p.757.

43 Rigby, M.J. (2019). Ethical dimensions of using artificial intelligence in health care. AMA Journal of Ethics. 21(2):E121‐124.

44 Recht, et al., op. cit. note 1, p.3582.

45 Publications Office of the European Union. (2019). Ethics guidelines for trustworthy AI. Retrieved May 24, 2023, from: https://data.europa.eu/doi/10.2759/346720.

46 Institute of Electrical and Electronics Engineers (IEEE). (2019). Ethically aligned design. First Edition Overview. A vision for prioritizing human well‐being with autonomous and intelligent systems. IEEE Global Initiative on Ethics of Autonomous and Intelligent Systems. Retrieved October 11, 2022, from https://sagroups.ieee.org/global-initiative/wp-content/uploads/sites/542/2023/01/ead1e-overview.pdf.

47 He, J., Baxter, S.L., Xu, J., Xu, J., Zhou, X., & Zhang, K. (2019). The practical implementation of artificial intelligence technologies in medicine. Nature Medicine. 25(1):30‐36.

48 Mittelstadt, B. (2021). The impact of artificial intelligence on the doctor–patient relationship. Council of Europe. Retrieved November 24, 2022, from https://www.coe.int/en/web/bioethics/report-impact-of-ai-on-the-doctor-patient-relationship.

49 Rudin, C. (2019). Stop explaining black‐box machine learning models for high‐stakes decisions and use interpretable models instead. Nature Machine Intelligence. 1(5):206‐215; Burrell, J. (2016). How the machine ‘thinks’: Understanding opacity in machine learning algorithms. Big Data & Society. 3(1):1‐12; Wadden, J.J. (2022). Defining the undefinable: The black box problem in healthcare artificial intelligence. Journal of Medical Ethics. 48:764‐768; High‐Level Expert Group on Artificial Intelligence. (2019). A definition of AI: Main capabilities and disciplines. Retrieved July 10, 2022, from https://ec.europa.eu/futurium/en/system/files/ged/ai_hleg_definition_of_ai_18_december_1.pdf

50 Wadden., op. cit. note 49, p.764.

51 Gilpin, L.H., Bau, D., & Yuan, B.Z., Bajwa, A., Specter, M., & Kagal, L. (2018). Explaining explanations: An overview of interpretability of machine learning. IEEE 5th International Conference on Data Science and Advanced Analytics.

52 Adadi, A., & Berrada, M. (2018). Peeking inside the black‐box: A survey on explainable artificial intelligence (XAI). IEEE Access. 6:52138‐52160.

53 Republic of South Africa. (1996). Constitution of the Republic of South Africa, 1996. Retrieved January 14, 2023, from https://www.gov.za/documents/constitution/constitution-republic-south-africa-1996-1.

54 Republic of South Africa. (2000). Promotion of Access to Information Act 2 of 2000. Retrieved January 22, 2023, from https://www.gov.za/documents/promotion-access-information-act.

55 Republic of South Africa. (2008). Consumer Protection Act 68 of 2008. Retrieved January 26, 2023, from https://www.gov.za/documents/consumer-protection-act

56 Republic of South Africa. (2013) Protection of Personal Information Act 4 of 2013. Retrieved February 4, 2023, from https://www.gov.za/documents/protection-personal-information-act.

57 Health Profession Council of South Africa (HPCSA). (2016). General ethical guidelines for health care professions. Retrieved August 21, 2022, from https://aestheticdoctors.co.za/wp-content/uploads/2023/02/Booklet-2-Ethical-and-professional-rules-of-the-Health-Professions-of-South-Africa.pdf.

58 OECD, op. cit. note 2, p.8.

59 HPCSA, op. cit. note 57, p.90‐91.

60
*Castell v De Greef* 1994 (4) SA 408 (C).; *Christian Lawyers’ Association v National Minister of Health* [2004] 4 All SA 31 (T).

61 National Health Act 61 of 2003, op. cit. note 16.

62 HPCSA, op. cit. note 57, p.90‐91.

63 Promotion of Access to Information Act, op. cit. note 54.

64
*Christian Lawyers’ Association v National Minister of Health*., op. cit. note 60.

65 American College of Radiology Data Science Institute, op. cit. note 28.

66 United Nations Educational, Scientific and Cultural Organization (UNESCO). (2021). Recommendation on the Ethics of Artificial Intelligence. Paris: UNESCO. Retrieved August 26, 2022, from https://unesdoc.unesco.org/ark:/48223/pf0000381137.

67 Protection of Personal Information Act, op cit. note 56.

68 Ibid: 19.

69 Ibid: 18.

70 Donnelly, D‐L. (2020). Privacy by (re) design: A comparative study of the protection of personal information in the mobile applications ecosystem under United States, European Union and South African law. University of KwaZulu‐Natal ResearchSpace, Retrieved October 15, 2022, from https://researchspace.ukzn.ac.za/handle/10413/19431

71 Morley, J., Taddeo, M., & Floridi, L., (2019). Google Health and the NHS: Overcoming the trust deficit. The Lancet Digital Health. 1(8):e389; Powles, J., & Hodson, H. (2017). Google DeepMind and healthcare in an age of algorithms. Health and Technology. 7(4):351‐367.

72 World Health Organization. (2021). Ethics and governance of artificial intelligence for health. Guideline. Retrieved August 26, 2022, from https://www.who.int/publications/i/item/9789240029200.

73 Beauchamp, T. L., & Childress, J. F. (8th ed). (2019). Principles of biomedical ethics. (pp. 116‐118). New York, NY: Oxford University Press.

74 Lochard, J., & First Thomas, S. (2016). Tenforde Topical Lecture: The ethics of radiological protection. Health Physics. 110(2):201‐210.

75 Miles, S.H. (2005). The Hippocratic Oath and the ethics of medicine: New York: Oxford University Press.

76 National Health Act 61 of 2003, op. cit. note 16; Constitution of the Republic of South Africa, op. cit. note 53; HPCSA, op. cit. note 57.

77 UNESCO, op. cit. note 66, p.10.

78 OECD., op. cit. note 2, p.8.

79 Miller, D.L. & Schauer, D. (2015). The ALARA principle in medical imaging. American Association of Physicists in Medicine Newsletter. 44:595‐600. Retrieved June 27, 2022, from https://issuu.com/aapmdocs/docs/4001-aapmnews?mode=embed&viewMode=singlePage&backgroundColor=eeeeee.

80 Consumer Protection Act, op. cit. note 55; Giuffrida, I. (2019). Liability for AI decision‐making: Some legal and ethical considerations. Fordham Law Review. 88:439.

81 Price, W.N., Gerke, S., & Cohen, I.G. (2019). Potential liability for physicians using artificial intelligence. JAMA. 322(18):1765‐1766.

82 Consumer Protection Act, op. cit. note 55.

83 Ibid.

84
*Motus Corporation (Pty) Ltd and Another v Wentzel* (Case no 1272/2019) [2021] ZASCA 40.

85 Barnard, J. (2022). An overview of the Consumer Safety and Product Liability regime in South Africa. International Journal on Consumer Law and Practice. 9:25.

86 Consumer Protection Act, op. cit. note 55.

87
*Afrox Healthcare Bpk v Strydom* 2002 6 SA 21 (SCA).

88 Naude, T., & Lubbe, G. (2005). Exemption clauses – a rethink occasioned, *Afrox Healthcare Bpk v Strydom*. South African Law Journal. 122(2):441‐463.

89 McQuoid‐Mason, D. (2012). Hospital exclusion clauses limiting liability for medical malpractice resulting in death or physical or psychological injury: What is the effect of the Consumer Protection Act? South African Journal of Bioethics and Law. 5(2):65‐68.

90 Constitution of the Republic of South Africa, op. cit. note 53.

91 Donnelly, D. (2022). First do no harm: Legal principles regulating the future of artificial intelligence in health care in South Africa. Potchefstroom Electronic Law Journal. 25(1):1‐43.

92 Oosthuizen, H., & Verschoor, T. (2008). Ethical principles becoming statutory requirements: CPD. South African Family Practice. 50(5):36‐40.

93 Naidoo, et al., op. cit. note 27, p.15.

94 Ibid: 14.

95 Giuffrida, I. (2019). Liability for AI Decision‐Making: Some Legal and Ethical Considerations. 88 Fordham Law Review. 439; Lemley, M.A., & Casey, B. (2019). Remedies for robots. The University of Chicago Law Review. 86(5):1311‐1396.

96 Soyer, B., & Tettenborn, A. (2023). Artificial intelligence and civil liability – do we need a new regime? International Journal of Law and Information Technology. 30(4):385‐397.

97 Ibid: 389.

98 Ibid: 388.

99 IMDRF, op. cit. note 24, p.10‐14.; Evas, T., Siffert, J., Kasavetova, D., Stoyanova, D., Dipalo, S., Novakovic, M., et al. (2020). Civil liability regime for artificial intelligence: European added value assessment. European Parliament, Directorate‐General Research Services.

100 Health Service and Resources Administration. (2023). Countermeasures Injury Compensation Program (CICP) USA. Retrieved April 26, 2023, from https://www.hrsa.gov/cicp.

101 Naidoo, et al., op. cit. note 27, p.14.

102 WHO, op. cit. note 72, p.121.

103 Constitution of the Republic of South Africa, op. cit. note 53.

104 IMDRF, op. cit. note 5, p.10.

105 Republic of South Africa. (2000). The Promotion of Equality and Prevention of Unfair Discrimination Act 4 of 2000. Retrieved January 22, 2023, from https://www.gov.za/documents/promotion-equality-and-prevention-unfair-discrimination-act.

106 Koch, B., Denton, E., Hanna, A., & Foster, J.G. (2021). Reduced, reused and recycled: The life of a dataset in machine learning research. Proceedings of the Neural Information Processing Systems Track on Datasets and Benchmarks. 1:1‐13; Wen, D., Khan, S.M., Xu, AJ., Ibrahim, H., Smith, L., Caballero, J., et al. (2022). Characteristics of publicly available skin cancer image datasets: A systematic review. The Lancet Digital Health. 4(1):e64‐e74.

107 Gianfrancesco, M.A, Tamang, S., Yazdany, J., & Schmajuk, G. (2018). Potential biases in machine learning algorithms using electronic health record data. JAMA Internal Medicine. 178(11):1544‐1547.

108 Obermeyer, Z., Powers, B., Vogeli, C., & Mullainathan, S. (2019). Dissecting racial bias in an algorithm used to manage the health of populations. Science. 366(6464):447‐453.

109 Gichoya, J., Banerjee, I., Bhimireddy, AR., Burns, J.L., Celi, L.A., Chen, L.‐C., et al. (2022). AI recognition of patient race in medical imaging: A modelling study. The Lancet Digital Health. 4(6):e406‐e414.

110 Ibid: e412.

111 Geis, J.R, Brady, A.P, Wu, C.C., Spencer, J., Ranschaert, E., Jaremko, J.L., et al. (2019). Ethics of artificial intelligence in radiology: Summary of the Joint European and North American Multisociety Statement. Journal of the American College of Radiology. 16(11):1516‐1521.

112 WHO, op. cit. note 72, p.45.

113 Ibid: 84.

114 Owoyemi, A., Owoyemi, J., Osiyemi, A., & Boyd, A. (2020). Artificial intelligence for healthcare in Africa. Frontiers in Digital Health. 2:6.

115 Butcher, N., Wilson‐Strydom, M., Baijnath, M., Orlic, D., Smith, M., Neupane, B., & Shawe‐Taylor, J. (2021). Artificial Intelligence Capacity in Sub Saharan Africa‐Compendium Report. Artificial Intelligence for Development Africa.

116 Katurura, M.C., & Cilliers, L. (2018). Electronic health record system in the public health care sector of South Africa: A systematic literature review. African Journal of Primary Health Care & Family Medicine. 10(1):e1‐e8.

117 Publications Office of the European Union. (2022). Study on eHealth, interoperability of health data and artificial intelligence for health and care in the European Union: Final study report. Lot 2, Artificial Intelligence for health and care in the EU.

118 Owoyemi, et al., op. cit. note 114.

119 Mollura, D.J., Culp, M.P., Pollack, E., Battino, G., Scheel, J.R., Mango, V.L., Elahi, A., Schweitzer, A., & Dako, F. (2020). Artificial intelligence in low‐and middle‐income countries: innovating global health radiology. Radiology. 297(3):513‐520.

120 WHO. (2023). Regulatory considerations on artificial intelligence for health. World Health Organization. p.25. Retrieved November 20, 2023, from https://iris.who.int/handle/10665/373421.

121 Ibid: 25; Ciecierski‐Holmes, T., Singh, R., Axt, M., Brenner, S., & Barteit, S. (2022). Artificial intelligence for strengthening healthcare systems in low‐ and middle‐income countries: a systematic scoping review. npj Digital Medicine. 5(1):162, p. 6.

122 Kwong, J.C., Erdman, L., Khondker, A., Skreta, M., Goldenberg, A., McCradden, M.D., Armando J Lorenzo, A.J., & Rickard, M. (2022). The silent trial – the bridge between bench‐to‐bedside clinical AI applications. Frontiers in Digital Health. 16:4:929508; Truby, J., Brown, R.D., Ibrahim, I.A., & Parellada, O.C. (2022). A sandbox approach to regulating high‐risk artificial intelligence applications. European Journal of Risk Regulation. 13(2):270‐294.

123 Madiega, T.A. (2022). Artificial Intelligence Act and regulatory sandboxes. Think Tank European Parliament. Retrieved April 15, 2023, from https://www.europarl.europa.eu/thinktank/en/document/EPRS_BRI(2022)733544; Stahl, B.C., Antoniou, J., Bhalla, N., Brooks, L., Jansen, P., Lindqvist, B., et al. (2023). A systematic review of artificial intelligence impact assessments. Artificial Intelligence Review. 56, 12799–12831.

124 WHO, op. cit. note 72, p.86; Stahl, et al. op cit. note 115, p.12.

125 Geis, et al., op. cit. note 111, p.1518.

126 Lee, J., Lynch, C.A., Hashiguchi, L.O., Snow, R.W., Herz, N.D., Webster, J., Parkhurst, J., & Erondu, N.A. (2021). Interventions to improve district‐level routine health data in low‐income and middle‐income countries: a systematic review. BMJ Global Health. 6(6):e004223.

127 Mollura, et al., op. cit. note 119.

